# Molecular Detection and Characterization of Intestinal and Blood Parasites in Wild Chimpanzees (*Pan troglodytes verus*) in Senegal

**DOI:** 10.3390/ani11113291

**Published:** 2021-11-17

**Authors:** Pamela C. Köster, Justinn Renelies-Hamilton, Laia Dotras, Manuel Llana, Celia Vinagre-Izquierdo, Petras Prakas, Donatas Sneideris, Alejandro Dashti, Begoña Bailo, Marta Lanza, Alejandra Jiménez-Mejías, Carlota Muñoz-García, Aly S. Muadica, David González-Barrio, José M. Rubio, Isabel Fuentes, Francisco Ponce-Gordo, Rafael Calero-Bernal, David Carmena

**Affiliations:** 1 Parasitology Reference and Research Laboratory, Spanish National Centre for Microbiology, 28220 Majadahonda, Spain; pamelakster@yahoo.com (P.C.K.); dashti.alejandro@gmail.com (A.D.); BEGOBB@isciii.es (B.B.); mlanza@isciii.es (M.L.); alejandrajimenezmejias@gmail.com (A.J.-M.); carlota.mg114@gmail.com (C.M.-G.); muadica@gmail.com (A.S.M.); dgonzalezbarrio@gmail.com (D.G.-B.); jmrubio@isciii.es (J.M.R.); ifuentes@isciii.es (I.F.); 2 Section for Ecology and Evolution, Department of Biology, University of Copenhagen, DK-1165 Copenhagen, Denmark; claxon71@gmail.com; 3 Jane Goodall Institute Spain and Senegal, Dindefelo Biological Station, Dindefelo, Kedougou, Senegal; laia.dotras@janegoodall.es (L.D.); manuel.llana@janegoodall.es (M.L.); 4 Evolutionary Ecology, Biology Department, Lund University, 223 62 Lund, Sweden; celia.vinagre.izquierdo@gmail.com; 5 Nature Research Centre, LT-08412 Vilnius, Lithuania; prakaspetras@gmail.com (P.P.); donatas.sneideris@gmail.com (D.S.); 6 Departamento de Ciências e Tecnologia, Universidade Licungo, Quelimane 106, Mozambique; 7 Department of Microbiology and Parasitology, Faculty of Pharmacy, Complutense University of Madrid, 28040 Madrid, Spain; pponce@ucm.es; 8 Salud Veterinaria y Zoonosis (SALUVET), Department of Animal Health, Faculty of Veterinary, Complutense University of Madrid, 28040 Madrid, Spain

**Keywords:** protists, non-human primates, endangered, conservation, PCR, genotyping, epidemiology, zoonoses, seasonality

## Abstract

**Simple Summary:**

Western chimpanzees are currently listed as a Critically Endangered subspecies. Human encroachment has taken a toll on this great ape due to fragmented habitat and the exchange of pathogens. This epidemiological study investigated the occurrence and genetic diversity of intestinal and blood parasites in faecal samples from wild chimpanzees living in the Dindefelo Community Nature Reserve, Senegal. We paid special attention to potential human-driven sources of infection and transmission pathways. Potential diarrhoea-causing protist parasites (e.g., *Cryptosporidium* spp., *Giardia duodenalis*, *Entamoeba histolytica*) were detected at low infection rates (and densities) or absent, whereas commensals (*Entamoeba dispar*) or protist of uncertain pathogenicity (*Blastocystis* sp.) were far more abundant. We detected *Sarcocystis* spp. in chimpanzee faeces. Blood protist parasites such as *Plasmodium* spp. and *Trypanosoma brucei* spp. (the etiological agents of malaria and sleeping sickness, respectively, in humans) were also found at low prevalences, but microfilariae of the nematode *Mansonella perstans* were frequently found. Molecular analyses primarily revealed host-adapted species/genotypes and an apparent absence of gastrointestinal clinical manifestations in infected chimpanzees. Zoonotic events of still unknown frequency and directionality may have taken part between wild chimpanzees and humans sharing natural habitats and resources.

**Abstract:**

Wild chimpanzee populations in West Africa (*Pan troglodytes verus*) have dramatically decreased as a direct consequence of anthropogenic activities and infectious diseases. Little information is currently available on the epidemiology, pathogenic significance, and zoonotic potential of protist species in wild chimpanzees. This study investigates the occurrence and genetic diversity of intestinal and blood protists as well as filariae in faecal samples (*n* = 234) from wild chimpanzees in the Dindefelo Community Nature Reserve, Senegal. PCR-based results revealed the presence of intestinal potential pathogens (*Sarcocystis* spp.: 11.5%; *Giardia duodenalis*: 2.1%; *Cryptosporidium hominis*: 0.9%), protist of uncertain pathogenicity (*Blastocystis* sp.: 5.6%), and commensal species (*Entamoeba dispar*: 18.4%; *Troglodytella abrassarti*: 5.6%). *Entamoeba histolytica*, *Enterocytozoon bieneusi*, and *Balantioides coli* were undetected. Blood protists including *Plasmodium malariae* (0.4%), *Trypanosoma brucei* (1.3%), and *Mansonella perstans* (9.8%) were also identified. Sanger sequencing analyses revealed host-adapted genetic variants within *Blastocystis*, but other parasitic pathogens (*C. hominis*, *P. malariae*, *T. brucei*, *M. perstans*) have zoonotic potential, suggesting that cross-species transmission between wild chimpanzees and humans is possible in areas where both species overlap. Additionally, we explored potential interactions between intestinal/blood protist species and seasonality and climate variables. Chimpanzees seem to play a more complex role on the epidemiology of pathogenic and commensal protist and nematode species than initially anticipated.

## 1. Introduction

Western chimpanzee populations (*Pan troglodytes verus*) have decreased by 80% between 1990 and 2014 in West Africa [1]. A combination of anthropogenic (agriculture, poaching, extractive industries, infrastructure development, human-chimpanzee interactions, lack of law enforcement) and natural (infectious diseases) threats are putting chimpanzees at the brink of extinction [2]. The breaking down of natural barriers has increased the likelihood of cross-species transmission of several zoonotic viral (rabies virus, Herpes B virus, Ebola virus, Yellow fever virus), bacterial (*Campylobacter jejuni*, enterotoxigenic *Escherichia coli*, *Klebsiella pneumoniae*, *Streptococcus pneumoniae*, *Streptococcus pyogenes*, *Staphylococcus aureus*, *Mycobacterium tuberculosis*) and parasitic (*Trypanosoma* spp., *Toxoplasma gondii*, *Babesia* spp., Coccidia, nematodes, and cestodes) infections that pose a threat both to endangered species (such as chimpanzees) and humans living nearby [3].

Little information is currently available on the epidemiology and potential health impact of zoonotic intestinal and blood protist species in wild chimpanzee populations. These include diarrhoea-causing intestinal protist species such as *Cryptosporidium* spp., *Giardia duodenalis*, and *Entamoeba histolytica* [4,5,6,7], and the microsporidia *Enterocytozoon bieneusi* [8]. Other protist species have uncertain or limited pathogenic significance, such as the stramenopile *Blastocystis* sp. [9] or the ciliate *Balantioides coli* [10]. Non-pathogenic protists species include *Entamoeba dispar* or the ape-adapted ciliate *Troglodytella abrassarti* [11]. Regarding blood parasites, malaria infections by at least seven *Plasmodium* species exhibiting strict host specificity are known to circulate in African great apes [12]. Thus, *Plasmodium reichenowi*, *Plasmodium billcollinsi*, *Plasmodium billbrayi* and *Plasmodium gaboni* infect only chimpanzees, while *Plasmodium praefalciparum*, *Plasmodium blacklocki* and *Plasmodium adleri* are restricted to gorillas. However, recent molecular studies have demonstrated that transfer of *Plasmodium* from humans towards chimpanzees is possible under confinement conditions [13], raising the question of whether cross-species transmission is also feasible under natural environmental conditions. Similarly, wild chimpanzees have been demonstrated to be suitable hosts for *Trypanosoma brucei*, the etiological agent of human and animal African trypanosomiasis [14]. However, it should be noted that assessing the role of non-human primates (NHP) in the epidemiology of *Plasmodium* and *Trypanosoma* parasites is severely hampered by the intrinsic difficulty of obtaining blood and tissue samples from the investigated animal populations, particularly in natural habitats. This issue is extensive also to filarial nematode parasites, whose microfilarial stages are circulating in the blood stream of the infected host. In this regard, DNA from *Mansonella* spp. has been identified in chimpanzee faecal samples from Gabon and Cameroon [15].

The protist enteroparasites *Cryptosporidium* spp., *G. duodenalis*, *Blastocystis* sp., and the microsporidia *E. bieneusi* exhibit extensive intra-species genetic diversity, allowing the identification of several genotypes/subtypes with marked differences in host specificity and range. *Cryptosporidium* encompasses at least 45 valid species, with *C. hominis* and *C. parvum* causing most of the infections reported in humans and NHP globally [16]. *Giardia duodenalis* is currently regarded as a multi-species complex comprising eight (A to H) distinct assemblages, of which zoonotic assemblages A and B are commonly reported to infect humans and other mammal species [17]. At least 25 subtypes (ST) have been identified within *Blastocystis* sp., of which ST1–9 and ST12 have been reported in humans [18]. Finally, more than 500 *E. bieneusi* genotypes have been defined and grouped in 11 phylogenetic groups. Group 1 and Group 2 include most of the potentially zoonotic genotypes, whereas the rest of the clusters display genotypes with strong host specificity [8].

Genotyping and subtyping methods are central in epidemiological studies to trace the origin of infections, understand the circulation of the pathogen in particular populations and geographical areas, provide information on transmission pathways, and assess the occurrence and directionality of potential zoonotic events. Data from this molecular-based epidemiological study aims to investigate the occurrence and genetic diversity of intestinal and blood parasites in faecal samples from wild chimpanzees living in the Dindefelo Community Nature Reserve (hereafter, Dindefelo) in Senegal, with special attention to potential human-driven sources of infection and cross-species transmission pathways.

## 2. Materials and Methods

### 2.1. Study Area

We collected faecal samples in Dindefelo, located in the south-eastern part of the Kedougou region, Senegal. This area is Sudano-Guinean woodland savannah mosaic intermixed with agricultural fields [19,20]. This highly seasonal habitat has a long dry season that lasts from November to May. Over 7000 people live in and around the reserve in 14 villages and hamlets [21]. In this anthropogenic landscape, the chimpanzees compete with the human population for food resources [22], making this area particularly well suited for transmission dynamics studies between humans and wild chimpanzees. The water scarcity during the dry season causes humans, livestock and wildlife, including chimpanzees, to use some of the same water sources [19].

### 2.2. Sampling and Data Collection

We collected 234 fresh (<24 h old) faecal samples of wild chimpanzee living in sympatry with humans. We were helped by experienced local field assistants to opportunistically identify and sample between November 2018 and March 2020. We obtained 5‒10 g from the centre of faeces with sterile cotton swabs and stored them in 70% ethanol for preservation and transport to the lab. For each sample, we estimated the time in hours since defecation and noted faecal consistency using the Bristol Stool Scale (BSS), ranging 1‒7 [23]. Additionally, we obtained daily rainfall data (GPM-3IMERGDF) [24] and surface air temperature (AIRS3STD; AIRS Science Team) [25] estimates from satellite data and averaged them across the 14,000 hectare study area. For rainfall data, we created a cumulative rainfall variable by adding the precipitation data from the 10 days before sampling.

### 2.3. DNA Extraction and Purification

We isolated genomic DNA from about 200 mg of each faecal specimen of chimpanzee by using the QIAamp DNA Stool Mini Kit (Qiagen, Hilden, Germany) according to the manufacturer’s instructions, except that samples mixed with InhibitEX buffer were incubated for 10 min at 95 °C. Extracted and purified DNA samples were eluted in 200 μL of PCR-grade water and kept at 4 °C until further molecular analysis. We included a water extraction control in each sample batch processed.

### 2.4. Molecular Detection of Cryptosporidium spp.

We assessed the presence of *Cryptosporidium* spp. using a nested-PCR protocol to amplify a 587 bp fragment of the *ssu* rRNA gene of the parasite [26]. Amplification reactions (50 μL) included 3 μL of DNA sample and 0.3 μM of the primer pairs CR-P1/CR-P2 in the primary reaction and CR-P3/CPB-DIAGR in the secondary reaction (Table S1). Both PCR reactions were carried out as follows: one step of 94 °C for 3 min, followed by 35 cycles of 94 °C for 40 s, 50 °C for 40 s and 72 °C for 1 min, concluding with a final extension of 72 °C for 10 min. Sub-typing of the isolates identified as *C. hominis* was attempted at the *gp60* gene using the AL-3531/AL-3535 and AL-3532/AL-3534 primer pairs [27].

### 2.5. Molecular Differential Detection of Entamoeba histolytica and Entamoeba dispar

We carried out detection and differential diagnosis between pathogenic *E. histolytica* and non-pathogenic *E. dispar* by a qPCR method targeting a 172 bp fragment of the gene codifying the *ssu* rRNA gene of *E. histolytica* and *E. dispar* [28,29]. Amplification reactions (25 μL) consisted of 3 μL template DNA, 12.5 pmol of the primer set Ehd-239F/Ehd-88R, 5 pmol of each specific TaqMan^®^ probe (Table S1), and TaqMan^®^ Gene Expression Master Mix (Applied Biosystems). Detection of parasitic DNA was performed on a Corbett Rotor GeneTM 6000 real-time PCR system (QIAGEN) using an amplification protocol consisting of an initial hold step of 2 min at 55 °C and 15 min at 95 °C followed by 45 cycles of 15 s at 95 °C and 1 min at 60 °C. We included molecular biology grade water (no-template, negative Nzytech, Lisbon, Portugal) and genomic DNA (positive) controls in each PCR run.

### 2.6. Molecular Detection and Characterization of Giardia Duodenalis

We conducted *G. duodenalis* DNA detection using a real-time PCR (qPCR) method targeting a 62 bp region of the gene codifying the small subunit ribosomal RNA (*ssu* rRNA) of the parasite [30]. Amplification reactions (25 μL) consisted of 3 μL of template DNA, 0.5 µM of each primer Gd-80F and Gd-127R, 0.4 µM of probe (Table S1), and 12.5 μL TaqMan^®^ Gene Expression Master Mix (Applied Biosystems, Waltham, MA, USA). Cycling conditions and data analysis were as described above for the detection of *E. histolytica*/*E. dispar*.

We subsequently assessed *G. duodenalis* isolates that tested positive by qPCR by sequence-based multi-locus genotyping of the genes encoding for the glutamate dehydrogenase (*gdh*) [31], β-giardin (*bg*) [32], and triose phosphate (*tpi*) [33] proteins of the parasite. We conducted amplifications by semi-nested and nested PCR protocols using specific primer pairs (Table S1).

### 2.7. Molecular Detection of Sarcocystis spp.

We detected *Sarcocystis* spp. by nested PCR amplifying a 550–600 bp fragment of the *ssu* rRNA gene. Final volumes (25 µL) of reaction mixtures included 3 µL of DNA sample and 0.4 µM of the outer Sgrau183/Sgrau182 and the inner Spri1/Spri2 primer sets (Table S1). Cycling conditions were as follows: enzyme activation at 94 °C for 5 min, followed by 30 cycles of 94 °C for 45 s, 59 °C for 50 s and 72 °C for 1 min, concluding with a final extension step of 72 °C for 10 min. PCR conditions for primary and secondary reactions were the same.

### 2.8. Molecular Detection and Characterization of Blastocystis sp.

We identified *Blastocystis* sp. by a direct PCR protocol targeting the *ssu* rRNA gene of the parasite [34]. The assay uses the pan-*Blastocystis*, barcode primer pair RD5/BhRDr to amplify a PCR product of ~600 bp. Amplification reactions (25 μL) included 5 μL of template DNA and 0.5 μM of each primer (Table S1). Amplification conditions consisted of one step of 95 °C for 3 min, followed by 30 cycles of 1 min each at 94, 59 and 72 °C, with an additional 2 min final extension at 72 °C.

### 2.9. Molecular Detection and Characterization of Enterocytozoon bieneusi

We conducted *E. bieneusi* detection by a nested PCR protocol to amplify the internal transcribed spacer (ITS) region as well as portions of the flanking large and small subunit of the ribosomal RNA gene as previously described [35]. We used the outer EBITS3/EBTIS4 and inner EBITS1/EBITS2.4 primer sets (Table S1) to generate a final PCR product of 390 bp, respectively. PCR reactions (50 μL) consisted of 1 μL of template DNA and 0.2 μM of each primer. Cycling conditions for the primary PCR consisted of one step of 94 °C for 3 min, followed by 35 cycles of amplification (denaturation at 94 °C for 30 s, annealing at 57 °C for 30 s, and elongation at 72 °C for 40 s), with a final extension at 72 °C for 10 min. Conditions for the secondary PCR were identical to the primary PCR except only 30 cycles were carried out with an annealing temperature of 55 °C.

### 2.10. Molecular Detection of Balantioides coli

We attempted *B. coli* detection by a direct PCR assay to amplify the complete ITS1–5.8s-rRNA–ITS2 region and the last 117 bp (3′ end) of the *ssu*-rRNA sequence of this ciliate using the primer set B5D/B5RC [36]. PCR reactions (25 μL) consisted of 2 μL of template DNA and 0.4 μM of each primer (Table S1). PCR conditions were as follows: 94 °C for 10 min; 30 cycles of 94 °C for 1 min, 60 °C for 1 min, 72 °C for 1 min, and a final extension for 5 min at 72 °C.

### 2.11. Molecular Detection of Troglodytella spp.

We carried out this ciliate mutualist’s detection by a direct PCR method targeting a 401 bp fragment of the ITS region of the rDNA (ITS1-5.8S rDNA-ITS2) [37]. PCR reactions (25 µL) contained 2 µL of template DNA and 0.8 µM of each primer SSU-end/LSU-start (Table S1). Conditions of PCR for ITS amplification were initial denaturation for 2 min at 94 °C, 35 cycles of 45 s at 94 °C, 45 s at 50 °C, and 90 s at 72 °C, and terminal elongation for 5 min at 72 °C.

### 2.12. Molecular Detection of Plasmodium spp.

We performed *Plasmodium* spp. detection using a qPCR protocol targeting the *ssu* rRNA. Expected amplicon sizes varied depending on the species of *Plasmodium*. In the case of those infective to humans, the range would be from 356 bp for *P. falciparum* to 417 bp for *P. malariae.* The PCR mixture consisted of 1× Quantimix Easy Probes (Biotools, B&M Labs SA, Madrid, Spain), 0.2 μM of each primer (universal primer forward JM-U-0011-L and *Plasmodium*-specific reverse primer PLR-1080, Table S1), 0.2 × EvaGreen^®^ Dye (Biotium, Inc. Hayward, Fremont, CA, USA) and 5 μL of template DNA in a reaction volume of 20 μL. We used a Rotor-Gene Q (QIAGEN) to perform the amplification, beginning with 3 min at 95 °C, followed by 45 cycles of 15 s at 95 °C, and 45 s at 62 °C. A final step of the melt program consisting of stepwise temperature increases of 0.5 °C from 60 °C to 95 °C with fluorescence acquisition at each temperature transition was included. We determined positive samples by post-reaction analysis by melting temperature (Tm) curve (Tm = 77.5 °C ± 1.0 °C) and by 2% agarose or automatic gel electrophoresis and confirmed by sequencing.

### 2.13. Molecular Detection of Trypanosomatid Species

We performed trypanosomatidae detection using a qPCR targeting the *ssu* rRNA. Expected amplicon sizes varied depending on the species, being 521 bp for *T. brucei.* The PCR mixture consisted of 1× Quantimix Easy Probes (Biotools), 0.2 μM of each primer (universal primer forward JM-U-0011-L and trypanosomatidae-specific reverse primer JM-T-0012n-R, Table S1), 0.2 × EvaGreen^®^ Dye (Biotium) and 5 μL of template DNA in a reaction volume of 20 μL. Cycling conditions, melt step, and electrophoretic procedures were as described in Section 2.12.

### 2.14. Molecular Detection of Filarial Species

We detected filarial species using a qPCR targeting the ITS1 region of the main filarial species infecting humans [38]. Expected amplicon sizes varied depending on the filarial species; within the genus *Mansonella*, the range would be from 250 bp for *M. streptocerca* to 312 bp for *M. perstans*. The PCR mixture consisted of 1 × Quantimix Easy Probes (Biotools), 0.2/0.375 μM of the primer set FIL2-F/FIL 2-Loa/FIL2-R (Table S1), 0.2 × EvaGreen^®^ Dye (Biotium) and 5 μL of template DNA in a reaction volume of 20 μL. Conditions of PCR for ITS1 amplification were initial denaturation for 3 min at 95 °C, followed by 45 cycles of 10 s at 95 °C, 15 s at 50 °C, and 15 s at 60 °C. A final step of melt program consisting of stepwise temperature increases of 0.5 °C from 60 °C to 95 °C with fluorescence acquisition at each temperature transition was included. We determined positive samples by post-reaction analysis by melting temperature (Tm) curve (Tm = 77.5 ± 1.0 °C) and by 2% agarose or automatic gel electrophoresis and confirmed by sequencing (see below).

### 2.15. Sequencing

We directly sequenced positive-PCR/qPCR products of the expected sizes in both directions using appropriate primer sets (Table S1). We used capillary DNA sequencing electrophoresis using the BigDye^®^ Terminator chemistry (Applied Biosystems) on an ABI PRISM 3130 automated DNA sequencer.

We have deposited the sequences obtained in this study in GenBank under accession numbers MZ182323–MZ182324 (*C. hominis*), MZ182325–MZ182328 (*Blastocystis* sp.), MZ182329–MZ182352 (*Sarcocystis* spp.), MZ224016 (*T. abrassarti*), MZ272002 (*P. malariae*), MZ272003 (*Plasmodium* sp.), MZ272004–MZ272006 (*T. brucei*), and MZ285880–MZ285897 (*M. perstans*).

### 2.16. Phylogenetic Analysis

We inferred evolutionary relationships among the identified *Blastocystis* subtypes by a phylogenetic analysis using the neighbor-joining method in MEGA 6 [39]. The evolutionary distances were computed using the Kimura 2 parameter method and modelled with a gamma distribution. We estimated the reliability of the phylogenetic analyses at each branch node by the bootstrapping 1000 times. We retrieved *Blastocystis* sp. sequences identified in captive or free-living chimpanzees globally from the NCBI database and included in the phylogenetic analysis for reference and comparative purposes.

### 2.17. Statistical Analyses

We analysed the relationship between cumulative rainfall and temperature (independent variables) and each parasite’s presence (for parasites with >5 positive samples; dependent variable) with generalized linear models using a binomial family (glm function in stats package v.3.6.1) [40].

Additionally, we divided the year into two main seasons: dry season (November through May) and rainy season (June through October). Again, we analysed the relationship between seasonality and parasite presence with generalized linear models in the same way as above.

We analysed the relationship between BSS (independent variable) and each parasite’s presence (for parasites with >5 positive samples; dependent variable) with additional binomial generalized linear models (glm function in stats package v.3.6.1) [40]. Finally, we correlated rainfall and temperature with stool consistency by using a Spearman correlation [41].

### 2.18. Parasite Interactions

We analysed the pairwise relationship between parasites (by fitting Fisher´s tests [42] for all parasites with at least 10 positive samples. We corrected for multiple testing by using the Benjamini and Hochberg method [43]. Further, we plotted a Principal Component Analysis (PCA) using the function prcomp from the stats R package v.3.6.1. There, we included minimum surface air temperature averaged over 10 days before sampling, and cumulative rainfall totalled over 10 days before sampling and binomial variables for the six most prevalent parasites.

## 3. Results

We show the full dataset indicating main features of chimpanzee faecal samples at the time of collection and PCR and sequencing results for the intestinal and blood parasite and commensal species investigated in the present study in Table S2.

### 3.1. Prevalence of Intestinal and Blood Parasites

We report the PCR-based prevalence rates of intestinal and blood parasite species identified in the wild chimpanzee population under investigation in Table 1. Among intestinal protists, the most common pathogenic protist identified was *Sarcocystis* spp. (11.5%), followed by *G. duodenalis* (2.1%), and *C. hominis* (0.9%). *Entamoeba histolytica* was not detected. We found non-pathogenic *E. dispar* in 18.4% of samples, the stramenopile *Blastocystis* sp. at a prevalence rate of 5.6%, whereas the microsporidia *E. bieneusi* was undetected. Among ciliates, we found the commensal *T. abrassarti* in 5.6% of samples, but none of them tested positive for *B. coli*. We detected the DNA of blood protists belonging to the genera *Plasmodium* (*P. malariae*) and *Trypanosoma* (*T. brucei*) at low (<2%) infection rates. Finally, the filarial nematode *Mansonella perstans* was identified in 9.8% of samples.

### 3.2. Molecular Characterization of Intestinal Protist Species

Sequence analyses revealed that the two *Cryptosporidium*-positive isolates identified in the present study corresponded to *C. hominis*. One of them had 100% homology with reference sequence AF108865, whereas the remaining isolate differed from it by a single nucleotide polymorphism (SNP) involving an ambiguous (double peak) site at position 805 (Table 2). Attempts to determine the genotype family of these isolates at the 60 kDa glycoprotein (*gp60*) locus repeatedly failed.

The five DNA isolates that yielded a positive result for *G. duodenalis* by qPCR generated cycle threshold (Ct) values ranging from 25.6–33.9 (median: 31.4). None of these isolates could be successfully amplified at the *gdh*, *bg*, or *tpi* loci, so the assemblage/sub-assemblage of the parasite involved in these infections remained unknown.

We detected DNA of *Sarcocystis* spp. in 27 samples. We obtained pure sequences in 24 cases, while we observed double peaks presumably indicating coexistence of two or more genetic variants of the parasite in three additional samples. One 526 bp sequence (MZ182329) showed 99.6–99.8% similarity with *S. alces* (EU282018, KF8312734) and up to 97% similarity with other *Sarcocystis* spp. Eight 552 bp sequences (MZ182330–MZ182337) shared 99.3–100% similarity with *S. gracilis* and <95% similarity with other *Sarcocystis* spp. Five 503 bp sequences (MZ182338–MZ182342) displayed 99.0–100% similarity with *S. morae* and <95% similarity with other *Sarcocystis* spp. Three 508 bp sequences (MZ182343–MZ182345) revealed highest similarity with *S. truncata* (98.1–100%) and *S. japonica* (97.7–99.6%). Five sequences (MZ182346–MZ182350) varying in length (503–509 bp) demonstrated highest similarity with *S. sinensis* (93.4–100%), *S. bovifelis* (96.1–99.8%) and *S. bovini* (96.7–99.2%). Finally, two 522 bp sequences (MZ182351–MZ182352) showed 97.1–99.6% and 98.1–99.6% similarity with *S. capracanis* and *S. tenella*, respectively. Thus, we identified at least six genetic variants of *Sarcocystis* genus in faecal samples of chimpanzees.

**Figure 1:**
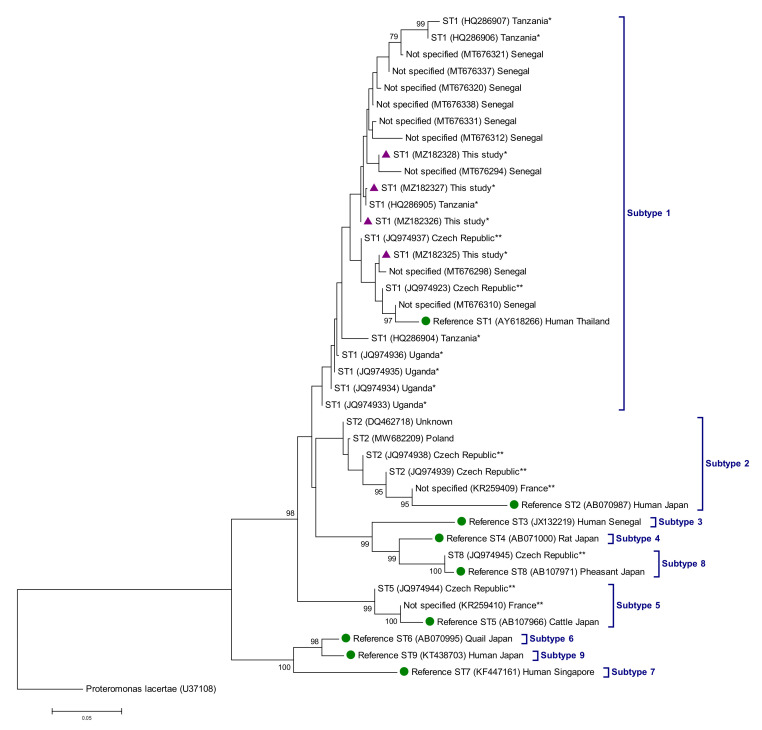
Phylogenetic relationships among *Blastocystis* sp. sequences identified in free-living and captive chimpanzees globally. GenBank accession numbers and country of origin are indicated. When known, sequences from free-living and captive chimpanzees were identified with superscript single and double asterisks, respectively. The analysis was conducted by a neighbor-joining method of the *ssu* rRNA gene. Genetic distances were calculated using the Kimura two-parameter model. Bootstrap values lower than 75% are not displayed. Purple-filled triangles represent sequences generated in the present study. Green filled dots represent reference sequences for subtypes ST1–ST9. *Proteromonas lacertae* was used as outgroup taxon to root the tree.

We confirmed a total of 13 isolates as *Blastocystis*-positive by Sanger sequencing. All of them were assigned to the subtype ST1 of the protist (Table 2). Allele 8 was the most common genetic variant found within ST1 (76.9%, 10/13), followed by allele 1 (7.7%, 1/13), allele 7 (7.7%, 1/13), and mixed alleles 7 + 8 (7.7%, 1/13). Phylogenetic analyses based on the neighbor-joining method confirmed that all ST1 sequences generated in the present study clustered together in well-supported clades with sequences of free-living chimpanzees from Senegal and Tanzania previously deposited in GenBank (Figure 1). Of note, an additional 42 isolates yielded amplicons of the expected size but in the form of faint bands on gel that produced poor quality, unreadable sequences. In the absence of Sanger sequencing confirmation, we conservatively considered these isolates *Blastocystis*-negative.

All 13 isolates yielding amplification products compatible with *T. abrassarti* were confirmed by Sanger sequencing. Representative sequences varied by two SNPs (T82C and G177A) with reference sequence EU680311 (Table 2).

### 3.3. Molecular Characterization of Blood Protist Parasites

Only two samples tested positive to *Plasmodium* spp. and an additional three to *T. brucei*. Plasmodia corresponded to *P. malariae* in one case and possibly also in the second (94% similarity with GenBank reference sequence XR_003751948.1). Phylogenetic analysis using the neighbor-joining method confirmed that both sequences belonged to the same cluster, although they were located in different tree branches (Figure S1). In the case of *T. brucei*, the three sequences generated were identical and fell into a well-defined cluster together with representative sequences of *T. brucei* (*T. brucei brucei*, *Tb rhodesiense*, *Tb gambiense*), *T. evansi*, and *T. equiperdum* retrieved from GenBank (Figure S2). Members of the above-mentioned species were integrated into the so-called brucei group [44]. The partial sequence of the *ssu* rRNA gene amplified by the PCR protocol used in the present study corresponded to a conserved region of the gene that does not allow for inter-species discrimination.

Sequence analyses of an additional 31 samples allowed the identification of other Trypanosomatida that are not considered parasites of primates. These include members of the genera *Phytomonas* (*n* = 28), *Herpetomonas* (*n* = 2) and *Lafontella* (previously *Herpetomonas*, *n* = 1), in addition to five cases of free-living Metakinetoplastin microorganisms of the orders Eubodonida (*n* = 2) and Neobodonida (*n* = 3). An ecological and epidemiological interpretation of these findings is provided in Table S1 (see text box on the upper right corner of the table).

### 3.4. Molecular Characterization of Filarial Parasites

We characterized 23 filarial nematodes by PCR and Sanger sequencing. All cases were homologous (≥99%) to *M. perstans*, although the sequences are not all the same as can be seen in the corresponding phylogenetic tree (Figure S3). In this case, all of them are integrated into the *M. perstans* cluster, close to the *M. streptocerca* cluster and further away from *M. ozzardi*.

### 3.5. Seasonality Effects on Parasites

**Figure 2:**
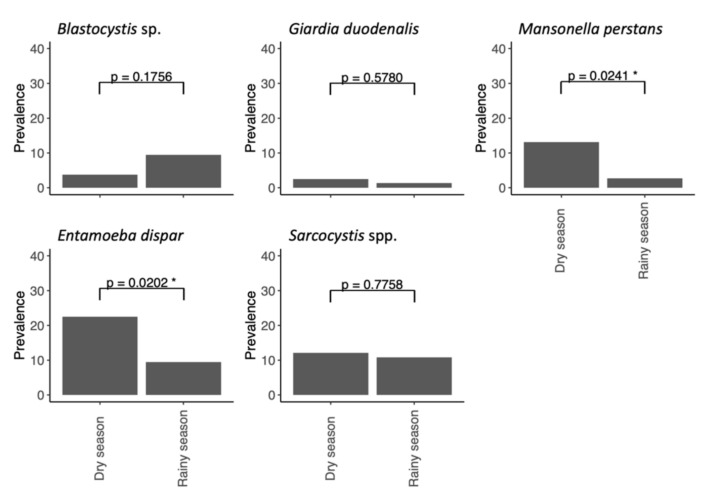
Chimpanzee parasite prevalence by season. Rainy season comprises months June through October, while dry season comprises November through May. See Table 3 for statistical differences between seasons. A superscript single asterisk denotes statistical significance.

Chimpanzee parasite prevalence differed by season (Figure 2; Table 3). *Mansonella perstans* had higher prevalence during the dry season than the rainy season (*p* = 0.0241), while the same is true for *E. dispar* (*p* = 0.0202).

**Figure 3:**
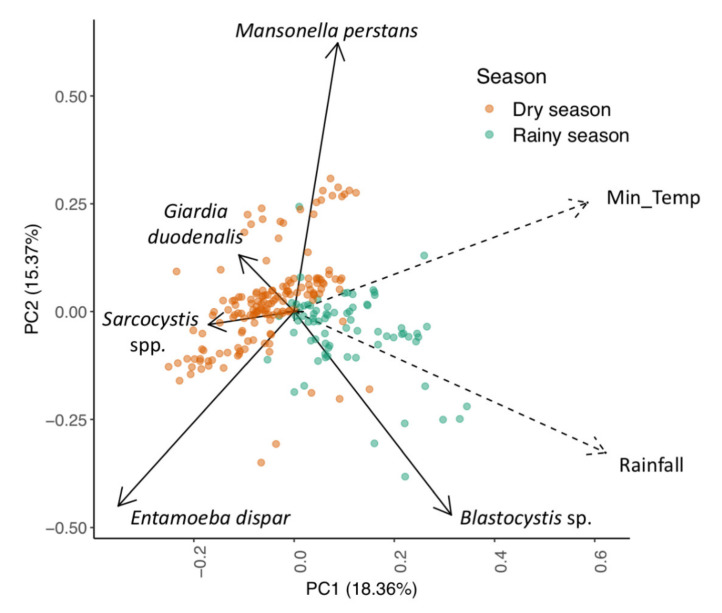
PCA plot of parasite presence in chimpanzee faecal samples. Each coloured dot indicates a single sample and is coloured by season. Solid arrows indicate parasite loading values, and dashed arrows indicate climate variable loading values. The closer two loading values are on the plot, the more likely they will correlate independently from other loading values. The distance from the coordinate centre (0, 0) can be used as a measure of strength of such associations. PC1 (positive values) captured high cumulative rainfall, while temperature was captured by a combination of PC1 and PC2 (positive values). Min_Temp represents minimum surface air temperature averaged over 10 days previous to sample collection. Rainfall represents cumulative rainfall totalled over the 10 days previous to sample collection.

Climate variables also explained the variance in parasite prevalence in chimpanzee samples (Figure 3): *Blastocystis* sp. (*p* = 0.0198) was positively associated with the total rainfall during the 10 days before sampling, whereas *M. perstans* (*p* = 0.0357) and *E. dispar* (*p* = 0.0066) were negatively associated with it. *Mansonella perstans* (*p* = 0.0213) and *Sarcocystis* spp. (*p* = 0.0338) were positively and negatively associated with the mean minimum temperature averaged over the 10 days before sampling, respectively.

While the Bristol Stool Scale (BSS) shows that rainfall and temperature cause faecal samples to become more watery (higher levels on the BSS), the faecal consistency did not significantly explain variance in any parasite’s prevalence (Table 3).

No negative or positive associations between parasites were detected (Table 4).

## 4. Discussion

In this PCR-based epidemiological study, we investigated the occurrence and genetic diversity of six potentially pathogenic (*C. hominis*, *E. histolytica*, *G. duodenalis*, *Sarcocystis* spp., *E. bieneusi*, and *B. coli*) and three non-pathogenic (*Blastocystis* sp., *E. dispar*, and *T. abrassarti*) intestinal protist and microsporidia species, two blood protist parasites (*Plasmodium* spp., and *Trypanosoma* spp.), and a filarial nematode (*M. perstans*) in faecal samples from a wild western chimpanzee population living in Dindefelo, Senegal. Overall, this is one of the most complete molecular-based surveys on protist parasite species affecting endangered chimpanzees conducted in Africa to date. The main contributions of this study to the field include (i) the finding, for the first time, of *Sarcocystis* spp. DNA in faecal samples of wild chimpanzees globally, (ii) the confirmation that common diarrhoea-causing protist species including *C. hominis*, *E. histolytica*, *G. duodenalis* or the microsporidia *E. bieneusi* are present at low infection rates and parasitic intensities or absent in the surveyed chimpanzee population, (iii) the confirmation that wild chimpanzees may act as suitable hosts of *Plasmodium* and *Trypanosoma* species and filariae, playing a still not fully understood role in their epidemiology and transmission [14,45,46], and (iv) the provision of epidemiological evidence in support of the existence of overlapping domestic and sylvatic cycles and cross-species transmission suggesting that some of the infections detected in chimpanzees might be of anthropogenic nature.

Little information is currently available on the epidemiology of *Cryptosporidium* spp. in wild chimpanzees. Early epidemiological studies based on microscopy examination failed to detect the parasite in the Republic of Congo [47], whereas a prevalence of 9% was found in savannah chimpanzees in Tanzania [48]. Using PCR, *Cryptosporidium* spp. was undetected in wild chimpanzees from the same study area as the present study in Senegal [49], whereas an infection rate of 21% was reported in chimpanzees from the Greater Gombe Ecosystem in Tanzania [50]. In the present study, only 1% of chimpanzee samples harboured *Cryptosporidium* infections. Regarding molecular diversity, Tanzanian wild chimpanzees were found to be infected by *C. hominis* (*n* = 6) and *C. suis* (*n* = 7) [50]. Remarkably, two of the six *C. hominis* isolates were identified as IfA12G2, the same sub-genotype detected in humans living in the proximity of the Gombe National Park. Of note, members of the *gp60* genotype family If have been found to be prevalent in different African countries including Kenya, South Africa, and Tanzania [51]. Taken together, these data strongly suggest that the *C. hominis* IfA12G2 infections detected in chimpanzees at the Greater Gombe Ecosystem were of anthropogenic origin [50]. In that very same study, the presence of *C. suis* in wild chimpanzees was interpreted as the result of cross-species transmission from bush pigs to chimpanzees in Gombe, since domesticated pigs were locally absent [50]. In the present study, the two *Cryptosporidium*-positive isolates were confirmed as *C. hominis*, but failure to amplify them at the *gp60* locus precluded us from identifying the *gp60* genotype family involved in those infections. More research should be conducted to elucidate the origin of these infections, as captive and wild NHP including chimpanzees can be infected by a wide range of *gp60* genotype families including Ia, Ib, Id, Ie, If, Ii, Ik, Im, and In [52].

*Entamoeba* spp. infection has been associated with morbidity and even mortality in NHP [53,54], although at present it remains to be fully elucidated if the *Entamoeba* species carried by NHP include the pathogenic *E. histolytica*. Indeed, *E. histolytica* was detected by PCR in wild and semi-wild orangutans in Sumatra, Indonesia [6]. In that study, contact with humans was considered as an important risk factor for infection of wild primates with this protist parasite. Molecular-based epidemiological surveys conducted in African wild chimpanzee populations have generated different, even discrepant, results. Thus, *E. histolytica* was undetected in populations, including those surveyed here, from Senegal [55] and Tanzania [56]. However, a subsequent study conducted in the latter country found an *E. histolytica* prevalence of 34% in chimpanzees from the Greater Gombe Ecosystem [57], whereas the protists were also present at low rates in chimpanzees living in the Dja Faunal Reserve in Cameroon [58]. These surveys coincided in proposing that interventions targeting better sanitation and hygiene practices for humans living in proximity with wild chimpanzee populations may help in preventing *E. histolytica* infection in NHP, while also protecting the endangered species.

The epidemiology of *G. duodenalis* in African wildlife is poorly understood. The few studies focusing on NHP, including lowland *(G. g. gorilla)* and mountain gorillas *(G. b. beringei)*, chimpanzees *(Pan troglodytes *schweinfurthii)**, baboons *(Papio anubis)*, greater bamboo lemurs (*Prolemur simus*) and eastern rufous mouse lemurs (*Microcebus rufus*), have been conducted in wildlife areas in Gabon, Madagascar, Rwanda, South Africa, and Tanzania [51]. In wild chimpanzee’s populations, *G. duodenalis* has been reported at infection rates of 3‒9% by immunofluorescence microscopy in the Republic of Congo [47], of 6% by light microscopy in the Cantanhez National Park in Guinea Bissau [59], and of 1% by PCR in Senegal [49]. In the latter study, the assemblage/sub-assemblage of the only *G. duodenalis*-positive isolate detected could not be determined due to insufficient amount of starting parasitic DNA. This is also the case in the present study, where the five samples positive to the protists failed to be amplified at the three loci (*gdh*, *bg*, *tpi*) used for genotyping purposes. These results strongly suggest low *G. duodenalis* intensity infections and, very likely, the absence of clinical manifestations in the infected chimpanzees. In other African wild NHP species, sub-assemblage AII has been identified in western lowland gorillas in the Central African Republic [60], assemblage A and sub-assemblage BIV in mountain gorillas in Rwanda [61] and Uganda [62], and sub-assemblages AII and BIV in colobus monkeys in Ghana [63] and Uganda [64]. Remarkably, several of these studies highlighted that anthropogenic habitat disturbance is enhancing interactions among people, livestock, and wildlife, and this could have negative consequences for wildlife conservation [61,63,64].

Experimental infections demonstrate that NHP, including chimpanzees, may serve as definitive hosts for zoonotic *Sarcocystis hominis* and *Sarcocystis suihominis* [65,66]. These coccidians are characterised by an obligatory prey–predator two-host life cycle. Definitive hosts become infected by ingesting extra-intestinal tissues (usually muscles) containing mature sarcocysts, while intermediate hosts become infected through ingestion of food or water contaminated with sporocysts [66]. In the present study, several different *Sarcocystis* genetic variants were confirmed in 11.5% (27/243) samples by nested PCR targeting *ssu* rRNA; obtained sequences displayed the highest genetic similarity with GenBank out-of-Africa available sequences of *Sarcocystis* spp. using Cervidae (absent in sub-Saharan Africa), Bovini (*Bos taurus*, present in the region) and Caprinae (*Capra* sp. Djallonké and *Ovis* sp. Djallonké, present in the region) as intermediate hosts [67,68,69]. Other antelopes in the region include *Cephalophus rufilatus* and *Tragelaphus scriptus*. There are three non-exclusive hypotheses for the presence of *Sarcocystis* spp. in chimpanzee faecal samples: (i) chimpanzees have been infected by consuming muscle tissue of some of these wild and domestic prey hosts, (ii) chimpanzees have acquired cysts from infected prey, but are not infected and do not represent definitive hosts for these parasites, and (iii) chimpanzees have acquired *Sarcocystis* spp. from the environment. Therefore, the results presented here do not serve as definitive evidence for these *Sarcocystis* spp. infecting chimpanzees, nor for locally novel prey items for chimpanzees. What is novel, to the best of our knowledge, is the presence of *Sarcocystis* genetic variants belonging to species considered to have Cervidae as intermediate hosts, mostly absent in the African continent [67].

*Blastocystis* sp. has been identified in more than 75 species of captive and wild NHP from 21 different countries. In free-living NHP, *Blastocystis* sp. infection/colonisation rates have been reported in the range of 22–100% globally [9]. Specifically, in African wild chimpanzee populations *Blastocystis* sp. has been documented at a prevalence of 22% by conventional microscopy in Cameroon [70], and of 41–71% by PCR in Senegal [49] and Tanzania [71]. The 6% prevalence rate reported here is much lower than those described above, but it should be noted that this figure is a conservative estimation as only isolates confirmed by Sanger sequencing were considered as true *Blastocystis*-positive samples. Regarding genetic diversity, ST1 is known to be by far the most prevalent (82–100%) *Blastocystis* subtype circulating in wild chimpanzees in Senegal and Tanzania, although in the former country few animals carried also ST2 and ST3 [49,71]. In line with these previous results, ST1 was also the only subtype detected in our chimpanzee population. Remarkably, sequence data revealed that all ST1 isolates detected involved allele 8 and, to a much lesser extent, alleles 1 and 7. In contrast, human infections/colonisation by ST1 are primarily due to allele 4 in both developed or developing countries [72,73]. Taken together, this data suggests that different host-adapted genetic variants of ST1 are circulating in human and NHP populations.

In this study, *E. bieneusi* was undetected in wild chimpanzees. Although this microsporidia has been reported in 32 species of NHP from 10 countries, most of these studies were conducted in captive animals. In wild NHP, *E. bieneusi* infection rates have been documented in the range of 3‒28% worldwide [8]. Regarding the presence of *E. bieneusi* in African chimpanzees, there is no information available in wild populations, but this parasite has been reported in chimpanzees living in sanctuaries in Kenya (3.6%) and Cameroon (4.5%) [74]. In other African wild NHP species, *E. bieneusi* has been identified at prevalence rates of 4.0% in western lowland gorillas (*G. g. gorilla*) in Central African Republic, of 12.3% in olive baboons (*P. anubis*) in Kenya, and of 18.0% in mountain gorillas (*G. b. beringei*) in Rwanda [8]. Further studies are needed to fully elucidate the role of wild chimpanzees in the transmission of *E. bieneusi*.

*Balantioides coli* presence has been reported in a wide range of host species, including chimpanzees [10]. It is considered a rare finding in wild populations; however, it is fairly common in captive animals. On the other hand, *T. abrassarti* is frequently reported in wild individuals but usually with low prevalence in captive ones [75,76,77,78,79]. While differences in diet have been suggested as an explanation for this pattern, the reasons behind it remain unclear [77,78]; other factors, such as the ground use by the chimpanzees and their interaction with human populations, could also explain differences in *B. coli* prevalence between captive and wild chimpanzees [75,80]. In the present work, the chimpanzee population under study was of wild animals; however, the study area is an anthropogenic landscape with chimpanzee–human-livestock interaction in the use of food and water resources [19,22]. The negative results obtained here can be explained by either absence of the parasite in human and livestock populations of the region, which should be confirmed by studying these host species, or by the fact these chimpanzees are wild, while more consistent and intense contact between humans and chimpanzees, such as what occurs in captivity, is required for zoonotic transmission. Equally surprising is the low prevalence of *T. abrassarti*, detected only in 5.6% of analysed samples. This ciliate does not form cysts and transmission is by ingestion of trophozoites contaminating food; they can be detected in faeces up to more than two days after defecation [81], which is twice the time after sampling collection in this survey. However, decomposition of trophozoites began immediately after defecation [81], so it is likely that DNA degradation under environmental conditions is responsible for the low PCR-based prevalence reported here. Despite *T. abrassarti* having a greater prevalence in wild chimpanzees, the colonisation density is apparently lower in wild chimpanzees than in captive ones [78]. This too has been considered a possible cause of negative results when searching for this ciliate in faecal samples [81]. Because of the above-mentioned limitations, data on the true prevalence of *T. abrassarti* should be interpreted with caution because the true prevalence of this ciliate is likely much higher. Moreover, we avoided analysing its seasonality, as it will only provide spurious results.

Faeces are not the optimal sample matrix for the detection of blood parasites such as *Plasmodium* or *Trypanosoma*, which are preferably identified in blood samples. However, several tests performed in humans [82] and NHP [83] have shown a good correlation between the diagnostic results obtained in dried blood spots and faecal samples, although large-scale field studies showed that the prevalence obtained was lower using faeces, at least in malaria [84]. In either case, the use of non-invasive, faecal-derived sampling methods, which can be implemented alongside routine monitoring and surveillance for other faecal parasites, gives it an added value, since otherwise these parasites would be difficult to identify in wild chimpanzee populations.

In this study, we detected five blood protist parasites: two *Plasmodium* spp. (probably both *P. malariae*) and three *T. brucei*. Several species of *Plasmodium* infect NHP exclusively, although they can sometimes cause sporadic human infections such as *P. cynomolgi* [85] or the most common *P. knowlesi* [86]. It is widely believed that human malaria parasites infect only people as a natural host, but many *Plasmodium* morphologically similar to those found in humans have been observed in NHP and, in certain cases, are even genetically identical. This is the case of *P. brasiliensis* in the Americas. In chimpanzees, *P. rodhaini* is morphologically identified as *P. malariae*-like parasite. Recent studies on African great apes have revealed the existence of a large diversity of *Plasmodium* parasites infecting chimpanzees and gorillas, some of them related to the deadliest human parasite *P. falciparum* (subgenus *Laverania*), others to the human parasites *P. malariae*, *P. ovale*, or *P. vivax* (subgenus *Plasmodium*) [87,88]. In this study, we identified a sample with *P. malariae*. A second sample could also be described as *P. malariae* according to phylogeny, as it fell within the same cluster of *P. malariae*/*P. brasiliensis*, although forming a different branch that could be considered as a *P. malariae*-like organism. Determining host specificity and host range of human malaria parasites is of great importance to understand the role of chimpanzees and other NHP in the epidemiology of the parasite, and to improve malaria control [89].

African trypanosomes of the *Trypanosoma brucei* group are the causative agents of sleeping sickness in humans and nagana in animals [90]. The group consists of seven species with the morphologically indistinguishable *T. b. rhodesiense* and *T. b. gambiense* causing the human African trypanosomiasis, and the rest being agents of infection in animals. In East Africa, animals are reservoirs for *T. b. rhodesiense*, whose infection produces nagana in them. However, in West Africa, where *T. b. gambiense* is primarily distributed, the role of animals as reservoirs of the parasite is not so clear [91]. In this study, the prevalence of *T. brucei* was low (1.3%). Of note, *T. brucei* has been previously reported in Western chimpanzees in Ivory Coast [14], but as in the case of our study, it was not possible to elucidate the subspecies present. In both cases, it was only possible to confirm their inclusion in the brucei group. As previous studies have shown, chimpanzees can become infected through the bites of the natural vector, tsetse fly (*Glossina* spp.), and complete the cycle, transmitting the disease to the vector [92]. Future studies determining the subspecies within the brucei group will be necessary to ascertain the frequency and directionality of zoonotic transmission events between chimpanzees and humans in endemic areas in West Africa. In this study, other trypanosomatidae, parasites of plants and insects, have been found in the faeces of the investigated chimpanzees, replicating the results obtained in earlier surveys [93]. These findings are possibly related to the chimpanzee diet or to the use that insects give to faeces to lay their eggs.

In this work, we have also detected DNA of filarial nematodes in chimpanzee faeces. Twenty-three samples (10%) were found to be positive, all of them characterized as *M. perstans* by phylogenetic analysis (Figure S3). Some of these sequences varied among them, forming a well-defined sub-cluster with a bootstrap support of 95%. In a recent study, *M. perstans* was found at a much lower frequency (2%) in chimpanzees from Cameroon and Gabon [15]. Furthermore, this survey provided sequences fitting within the same genus (*Mansonella*), but clustering in a different branch than that of *M. perstans*. In this case, the species could not be determined due to insufficient homology with previously described species. Unfortunately, in our study, we have not been able to compare the phylogeny of both groups of sequences as they correspond to different gene fragments. Some species of filariae, such as *Onchocerca volvulus* or *Wuchereria bancrofti*, have medical importance, although mansonellosis (the disease produced by *M. perstans*) is not considered a public health hazard [94]. The larvae of filariae (microfilariae) are found in the blood or the skin, while adults are found inside the host near the internal organs forming nodules or semi-protected remain confined in other areas. Therefore, the approach of attempting the detection of filarial DNA in faecal samples might seems unusual at first glimpse. In a seminal study, Gaillard et al. described for the first time the presence of *Mansonella* spp. DNA in faecal samples of infected chimpanzees [15]. These authors provided two potential explanations for this finding: (i) health issues associated with haematuria leading to the presence of blood parasites in the faeces of sick animals and (ii) free DNA or DNA included in exosomes secreted by the filarial parasite as mediators of the host–parasite interface. Consequently, the detection of these parasites in faeces is relevant to estimating prevalence rates without using invasive procedures to obtain other biological samples such as blood or tissues. The PCR-based method used here allowed for the detection of *M. perstans* (and possibly other filariae) present in chimpanzee faecal samples using pan-filarial primers regardless of species and hosts [38]. Additionally, this methodology can also provide relevant information on filariae abundance, pathogenesis, transmission and zoonotic potential in wild chimpanzees.

Data obtained here show that it is possible to detect *P. malariae*, *T. brucei*, and *M. perstans* in chimpanzee faecal material. These results suggest that stool samples are a promising, suitable matrix for the detection of blood parasites. However, we need to take into consideration that prevalence rates obtained in faecal samples do not necessarily reflect the ones we would identify in blood samples by conventional diagnostic methods. These three parasites normally infect humans and studying the role that chimpanzees can play as potential reservoirs would be important to optimize control programs for these diseases in areas where apes and humans live closely together.

Chimpanzee parasite seasonality has rarely been studied over more than a single dry or wet season [95,96,97,98,99]. Here, we aim to contrast results found in previous studies to our own. Beforehand, we must warn readers that independence of samples is not guaranteed in this study as individual chimpanzees could not be identified and results should, therefore, be interpreted with caution. *Blastocystis* was associated with rainfall, and undetected during the dry season in our study. One study in a degraded forest mosaic landscape in Uganda reports higher *Blastocystis* prevalence during the rainy season [95], and another report the same trend in a mixed evergreen and semi-deciduous forest habitat in Tanzania [96]. However, other studies report significant [98] and non-significant [98] associations between *Blastocystis* and the dry season. We found *Entamoeba dispar*’s prevalence to be higher during the dry season than the rainy season, and to be negatively associated with rainfall. However, previous studies reported a lack of association between *Entamoeba* spp. and seasonality [95,96,97]. Reports on *Giardia*’s seasonality do not find seasonal differences [96,98]. In them, however, prevalence was below 10%, as was the case in our study. Differences in prevalence rates according to season were also found for *M. perstans*, although absence of data in the literature precluded us from reaching strong conclusions. Further research, and in particular meta-analyses, are needed to clarify these trends, with particular focus on the factors that correlate with climate, rather than climate itself.

The results obtained and conclusions reached in this study may have been biased by some design and methodological constricts. For instance, collected faecal samples were stored in 70% ethanol at room temperature for several months before processing in the laboratory. This long storage period may have hampered the obtaining of sufficient amount of good quality parasitic DNA for PCR analyses. Low infection/colonisation rates for some of the parasite or commensal species investigated here may have negatively influenced the robustness of the statistical analyses conducted. Similarly, low parasitic densities (e.g., *G. duodenalis*, *C. hominis*) limited the performance of the PCR protocols used for genotyping and sub-genotyping purposes. Due to ethical and conservation concerns, no tissue or blood samples of animal origin were available for downstream molecular testing. In practical terms, this means that the occurrence of blood protist (*Plasmodium*, *Trypanosoma*) and filarial (*Mansonella*) species could only be indirectly assessed in faecal material, a sub-optimal matrix for the detection of these pathogens.

## 5. Conclusions

This molecular-based epidemiological study showed that potentially pathogenic protist/microsporidia species were present at low-medium infection rates (*Sarcocystis* spp., *G. duodenalis*, *C. hominis*) or apparently absent (*E. histolytica*, *E. bieneusi*) in a wild chimpanzee population in Dindefelo, Senegal. In contrast, protist of unclear pathogenic significance (*Blastocystis* sp.) and commensal species (*E. dispar*, *T. abrassarti*) were far more common. From a parasitological point of view, these data reflect an apparent healthy epidemiological situation characterised by low-to-moderate occurrence rates and parasitic burdens leading to absence of clinical manifestations in infected animals, although the extent of this claim should be confirmed in subsequent studies specifically devoted to assess the health status of these animals. The limited amount of parasitic DNA in faecal samples also explains the low amplification success rates accomplished by genotyping and sub-genotyping PCR protocols (e.g., for *G. duodenalis* and *C. hominis*, among others). Remarkably, this is the first survey reporting the presence of *Sarcocystis* spp. DNA in faeces from wild chimpanzees, raising the question of whether these findings are the result of true infections by this coccidian parasite or just spurious passage associated with diet or environmental contamination. Another major contribution of this survey is the demonstration that, even considering that faecal material is a suboptimal matrix for the detection of blood protist species, chimpanzees might be suitable hosts for *P. malariae* and *T. brucei*, two of the major contributors to the human disease burden by malaria and sleeping sickness in Africa. Overall, these data clearly indicate that wild chimpanzees play a more important role in the epidemiology and transmission of intestinal and blood protist parasites than initially anticipated. Taking into account that most of these agents are zoonotic, it is now clear that cross-transmission species is possible between wild chimpanzees and humans sharing habitats and natural resources. More research is needed to accurately assess the frequency and directionality of zoonotic events, and to estimate the proportion of chimpanzee infections of anthropogenic origin.

## Figures and Tables

**Table 1:** Prevalence of intestinal and blood parasite and commensal species identified in the wild chimpanzee population (*n* = 234) investigated in the present study.

Species	Positive (*n*)	Prevalence (%)	95% Confidence Interval
Intestinal protists			
*Entamoeba dispar*	43	18.4	13.6–23.9
*Sarcocystis* spp.	27	11.5	7.7–16.3
*Blastocystis* sp.	13	5.6	3.0–9.3
*Troglodytella abrassarti*	13	5.6	3.0–9.3
*Giardia duodenalis*	5	2.1	0.7–4.9
*Cryptosporidium hominis*	2	0.9	0.1–3.1
*Balantioides coli*	0	0.0	–
*Entamoeba histolytica*	0	0.0	‒
Intestinal microsporidia			
*Enterocytozoon bieneusi*	0	0.0	‒
Blood protists			
*Trypanosoma brucei*	3	1.3	0.27–3.7
*Plasmodium malariae*	1	0.4	0.01–2.4
*Plasmodium* spp.	1	0.4	0.01–2.4
Filarial nematodes			
*Mansonella perstans*	23	9.8	6.3–14.4

**Table 2:** Diversity, frequency, and molecular features of *Cryptosporidium hominis*, *Blastocystis* sp., and *Troglodytella abrassarti* isolates identified in the wild chimpanzee population investigated in the present study.

Species	Genotype	Sub- Genotype	No. Isolates	Locus	Reference Sequence	Stretch	Single Nucleotide Polymorphisms	GenBank ID
*Cryptosporidium hominis*	*‒*	*‒*	1	*ssu* rRNA	AF108865	574–997	None	MZ182324
	*‒*	*‒*	1	*ssu* rRNA	AF108865	579–983	C805Y	MZ182323
*Blastocystis* sp.	ST1	Allele 1	1	*ssu* rRNA	AB107968	79–568	A132G	MZ182325
	ST1	Allele 7	1	*ssu* rRNA	HQ286905	14–506	A476G	MZ182326
	ST1	Allele 8	10	*ssu* rRNA	HQ286907	1–553	T1A, 10_11DelAG, 71DelC, 513DelC, T551A, A553C	MZ182327
	ST1	Alleles 7 + 8	1	*ssu* rRNA	HQ286907	73–553	G141R, 513DelC, T551A, A553C	MZ182328
*Troglodytella abrassarti*	*‒*	*‒*	13	ITS	EU680311	1–418	T82C, G177A	MZ224016

Del: base deletion; ITS: Internal transcribed spacer; R: A/G; ssu rRNA: Small subunit ribosomal RNA; Y: C/T.

**Table 3:** Results of the statistical analyses conducted to assess the relationship between the occurrence of the most frequent parasite species infecting chimpanzees and climatological (rainfall, temperature) or clinical (stool consistency) variables in the present study. For seasonality, estimates are given on the log odds ratio (not the response) scale. Statistically significant values are highlighted in bold.

Variable	Contrast	Estimate	SE	Z Ratio	*p* Value
*Blastocystis* sp.	Seasonality	0.4770	0.3520	1.355	0.1756
	Rainfall	0.0548	0.0235	2.330	**0.0198**
	Temperature min.	−0.0263	0.2118	−0.124	0.9010
	Stool consistency	0.1069	0.1632	0.655	0.5120
*Giardia duodenalis*	Seasonality	0.6270	1.1300	0.556	0.5780
	Rainfall	−0.4564	0.4465	−1.022	0.3067
	Temperature min.	1.7000	0.0754	2.255	**0.0241**
	Stool consistency	−0.0967	0.0460	−2.100	**0.0357**
*Mansonella perstans*	Seasonality	0.3569	0.1588	2.303	**0.0213**
	Rainfall	−0.0435	0.0237	−1.838	**0.0066**
	Temperature min.	−0.1081	0.1177	−0.918	0.3590
	Stool consistency	−0.1626	0.1151	−1.412	0.1579
*Entamoeba dispar*	Seasonality	−1.4600	0.5770	−2.525	0.0570
	Rainfall	0.2614	0.1591	0.164	0.1000
	Temperature min.	0.1270	0.4470	0.285	0.7758
	Stool consistency	0.0228	0.0200	1.141	0.2538
*Sarcocystis* spp.	Seasonality	1.7000	0.0754	2.255	**0.0241**
	Rainfall	−0.2865	0.1756	−1.631	0.1028
	Temperature min.	1.0200	0.4400	2.323	**0.0202**
	Stool consistency	−0.0435	0.0237	−1.838	**0.0066**
Bristol Stool Scale	Seasonality	−1.6200	0.2230	−7.285	**<0.0001**
	Rainfall *	*t* = 5.961	df = 210	–	**<0.0001**
	Temperature min. *	*t* = 2.030	df = 210	–	**0.04361**

* Rainfall data/temperature and stool consistency (as determined using the Bristol Stool Scale) are correlated with a Spearman correlation.

**Table 4:** Results of the statistical analyses conducted to assess potential relationships between prevalent parasites.

Parasite 1	Parasite 2	*p* Value	*p* Adjusted Value
*Blastocystis* sp.	*Giardia duodenalis*	1.0000	1.0000
*Blastocystis* sp.	*Mansonella perstans*	1.0000	1.0000
*Blastocystis* sp.	*Entamoeba dispar*	0.2654	1.0000
*Blastocystis* sp.	*Sarcocystis* spp.	1.0000	1.0000
*Giardia duodenalis*	*Mansonella perstans*	1.0000	1.0000
*Giardia duodenalis*	*Entamoeba dispar*	0.5875	1.0000
*Giardia duodenalis*	*Sarcocystis* spp.	0.4660	1.0000
*Mansonella perstans*	*Entamoeba dispar*	0.0874	0.8740
*Mansonella perstans*	*Sarcocystis* spp.	0.7384	1.0000
*Entamoeba dispar*	*Sarcocystis* spp.	0.7934	1.0000

## Data Availability

All relevant data are within the article and its additional files. The sequences data were submitted to the GenBank database under the accession numbers MZ182323–MZ182324 (*C. hominis*), MZ182325–MZ182328 (*Blastocystis* sp.), MZ182329–MZ182352 (*Sarcocystis* spp.), MZ224016 (*T. abrassarti*), MZ272002 (*P. malariae*), MZ272003 (*Plasmodium* sp.), MZ272004–MZ272006 (*T. brucei*), and MZ285880–MZ285897 (*M. perstans*).

## Supplementary Materials

The following are available online at https://www.mdpi.com/article/10.3390/ani11113291/s1, Figure S1: Phylogenetic relationships among *Plasmodium* sequences identified in free-living chimpanzees in the present study and homologous sequences retrieved from GenBank. Sequences accession numbers are indicated. The analysis was conducted by a neighbor-joining method of the *ssu* rRNA gene using Treecon software [100] after ClustalW alignment of the sequences [101]. Bootstrap values lower than 75% are not displayed. *Haemosporidia* was used as outgroup taxon to root the tree, Figure S2: Phylogenetic relationships among *Trypanosoma* sequences identified in free-living chimpanzees in the present study and homologous sequences retrieved from GenBank. Sequences accession numbers are indicated. The analysis was conducted by a neighbor-joining method of the *ssu* rRNA gene using Treecon software [100] after ClustalW alignment of the sequences [101]. Bootstrap values lower than 75% are not displayed. *Trypanosoma godfreyi* was used as outgroup taxon to root the tree, Figure S3: Phylogenetic relationships among *Mansonella perstans* sequences identified in free-living chimpanzees in the present study and homologous sequences retrieved from GenBank. Sequences accession numbers are indicated. The analysis was conducted by a neighbor-joining method of the *ssu* rRNA gene using Treecon software [100] after ClustalW alignment of the sequences [101]. Bootstrap values lower than 75% are not displayed. *Loa loa* was used as outgroup taxon to root the tree. Table S1: Oligonucleotides used for the molecular identification and/or characterization of the intestinal and blood protist species investigated in the present study. Table S2: Full dataset indicating main features of chimpanzee faecal samples at the time of collection and PCR and sequencing results for the intestinal and blood protist species investigated in the present study.
