# Supplementary material for: Molecular Detection and Characterization of Intestinal and Blood Parasites in Wild Chimpanzees (Pan troglodytes verus) in Senegal
Source: Animals (Basel). 2021 Nov 17;11(11):3291. doi: 10.3390/ani11113291 (PMC8614354; doi:10.3390/ani11113291)
Supplement: Supplementary file 1 [file animals-11-03291-s001.zip › Figure S1 Manuscript Koster et al_Animals.pdf]

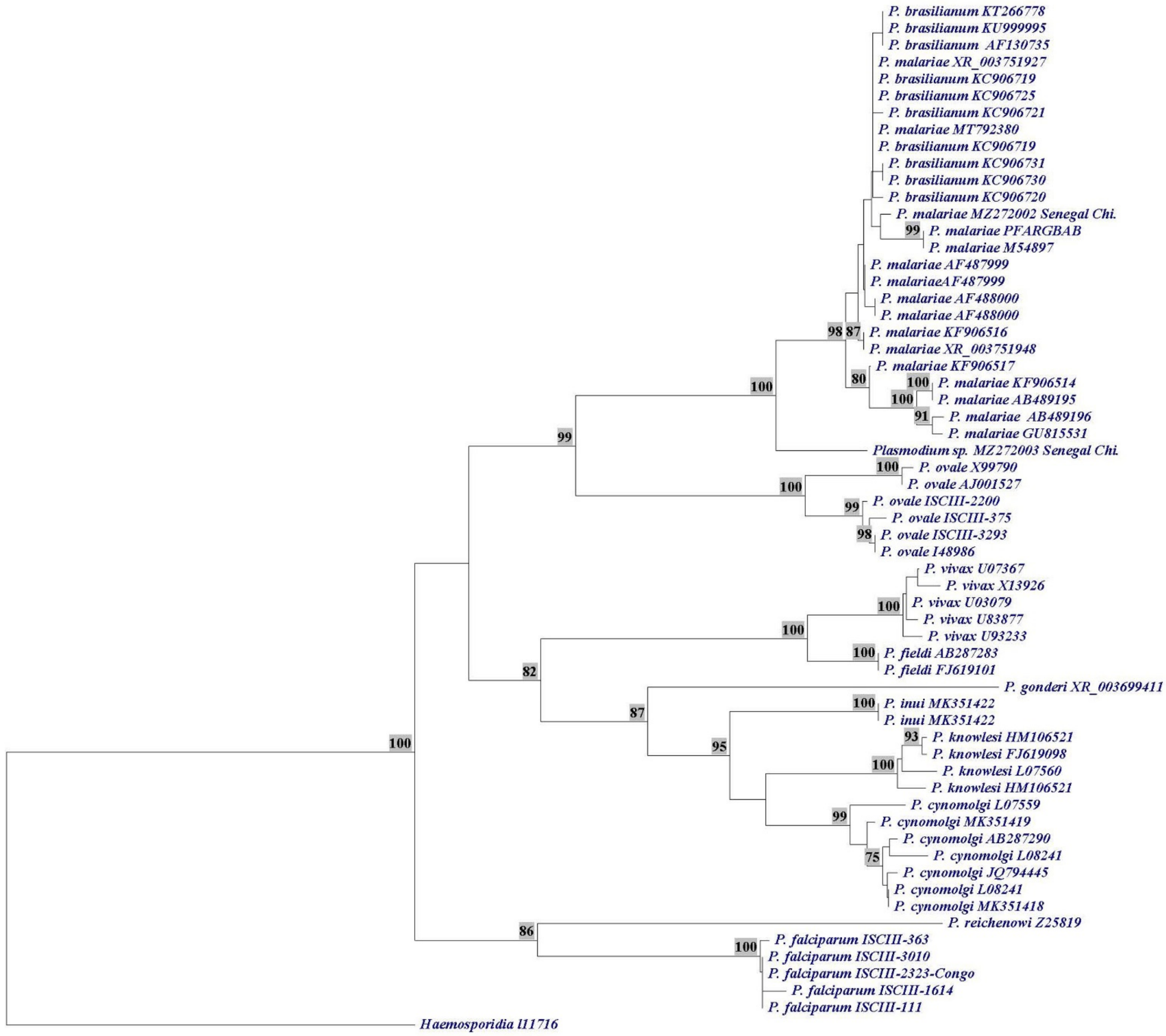

Figure S1. *Plasmodium* spp. *ssu* rRNA phylogenetic tree. Phylogenetic relationships of the *ssu* rRNA gene (partial fragment) among *Plasmodium* sequences identified in free-living chimpanzees in the present study (labeled as Senegal Chi), homologous sequences retrieved from GenBank (sequence accession numbers are indicated) and laboratory sequence database (ISCIII identification number). The analysis was conducted by the neighbor-joining method using Treecon software [96] after ClustalW alignment of the sequences [97]. Bootstrap values (1000 replicates) lower than 75% are not displayed. *Haemosporidia* was used as outgroup to root the tree.
