# Supplementary material for: Molecular Detection and Characterization of Intestinal and Blood Parasites in Wild Chimpanzees (Pan troglodytes verus) in Senegal
Source: Animals (Basel). 2021 Nov 17;11(11):3291. doi: 10.3390/ani11113291 (PMC8614354; doi:10.3390/ani11113291)
Supplement: Supplementary file 1 [file animals-11-03291-s001.zip › Table S2 Köster et al_Animals.pdf]

Table S2 Oligonucleotides used for the molecular identification and/or characterization of the enteric and hematic protist species investigated in the present study.

Target organism	Locus	Oligonucleotide	Sequence (5´–3´)	Reference
Cryptosporidium spp.	*ssu* rRNA	CR-P1	CAGGGAGGTAGTGACAAGAA	[23]
		CR-P2	TCAGCCTTGCGACCATACTC	[23]
		CR-P3	ATTGGAGGGCAAGTCTGGTG	[23]
		CPB-DIAGR	TAAGGTGCTGAAGGAGTAAGG	[23]
Entamoeba histolytica	*ssu* rRNA	Probe	FAM–TCATTGAATGAATTGGCCATTT–MGB	[24]
Entamoeba dispar		Probe	VIC–TTACTTACATAAATTGGCCACTTTG–MGB	[24]
Entamoeba histolytica/dispar		Ehd-239F	ATTGTCGTGGCATCCTAACTCA	[25]
Entamoeba histolytica/dispar		Ehd-88R	GCGGACGGCTCATTATAACA	[25]
Giardia duodenalis	*ssu* rRNA	Probe	FAM–CCCGCGGCGGTCCCTGCTAG–BHQ1	[26]
		Gd-80F	GACGGCTCAGGACAACGGTT	[26]
		Gd-127R	TTGCCAGCGGTGTCCG	[26]
	gdh	GDHeF	TCAACGTYAAYCGYGGYTTCCGT	[27]
		GDHiF	CAGTACACCTCYGCTCTCGG	[27]
		GDHiR	GTTRTCCTTGCACATCTCC	[27]

	bg	G7_F	AAGCCCGACGACCTCACCCGCAGTGC	[28]
		G759_R	GAGGCCGCCCTGGATCTTCGAGACGAC	[28]
		G99_F	GAACGAACGAGATCGAGGTCCG	[28]
		G609_R	CTCGACGAGCTTCGTGTT	[28]
	tpi	AL3543	AAATIATGCCTGCTCGTCG	[29]
		AL3546	CAAACCTTITCCGCAAACC	[29]
		AL3544	CCCTTCATCGGIGGTAACTT	[29]
		AL3545	GTGGCCACCACICCCGTGCC	[29]
Sarcocystis spp.	*ssu* rRNA	Sgrau183	TGGATAACCGTGGTAATTCTATG	Present study
		Sgrau182	TCGCAGTAGTTCGTCTTTAACAAA	Present study
		Spri1	GGCGATAGATCATTCAAGTTTCT	Present study
		Spri2	CCGAAATCCTATATTGTTATTCCAT	Present study
Blastocystis spp.	*ssu* rRNA	BhRDr	GAGCTTTTTAACTGCAACAACG	[30]
		RD5	ATCTGGTTGATCCTGCCAGT	[30]
Enterocytozoon bieneusi	ITS	EBITS3	GGTCATAGGGATGAAGAG	[31]
		EBITS4	TTCGAGTTCTTTCGCGCTC	[31]
		EBITS1	GCTCTGAATATCTATGGCT	[31]
		EBITS2.4	ATCGCCGACGGATCCAAGTG	[31]

Balantioides coli	ITS	B5D	GCTCCTACCGATACCGGGT	[32]
		B5RC	GCGGGTCATCTTACTTGATTTC	[32]
Troglodytella spp.	ITS	SSU_end)	AAGGTWTCCGTAGGTGAACCTG	[33]
		LSU_start	TAKTRAYATGCTTAAGTYCAGCG	[33]
Plasmodium spp.	*ssu* rRNA	JM-U-0011-L	CAAGTCTGGTGCCAGCAS	Present study
		PLR-1080	AARCTACTCCTATTAATCGTAACTAAGC	Present study
Trypanosoma spp.	*ssu* rRNA	JM-U-0011-L	CAAGTCTGGTGCCAGCAS	Present study
		JM-T-0012n-R	GAGAACGTACTGGTGCGTCGG	Present study
		JM-T-0013-S	GGCCACCGTTTCGGCTT	Present study
Filarioidea [1]	ITS-1	FIL2-F	GGTGAACCTGCGGAAGGATC	[34]
		FIL2-LOA	GGTGAACCTGCRGMWGGATC	[34]
		FIL2-R	TGCTTATTAAGTCTACTTAA	[34]

[1] Filarioidea superfamily includes members of the genera *Brugia*, *Dirofilaria*, *Loa*, *Mansonella*, *Onchocerca*, and *Wuchereria*.

bg: β-giardin (bg); *gdh*: Glutamate dehydrogenase; ITS: Internal transcribed spacer; *ssu* rRNA: Small subunit ribosomal RNA; *tpi*: Triose phosphate isomerase.
