# Supplementary material for: Molecular Detection and Characterization of Intestinal and Blood Parasites in Wild Chimpanzees (Pan troglodytes verus) in Senegal
Source: Animals (Basel). 2021 Nov 17;11(11):3291. doi: 10.3390/ani11113291 (PMC8614354; doi:10.3390/ani11113291)
Supplement: Supplementary file 1 [file Figure S2 Manuscript Koster et al_Animals.pdf › Figure S2 Manuscript Koster et al_Animals.pdf]

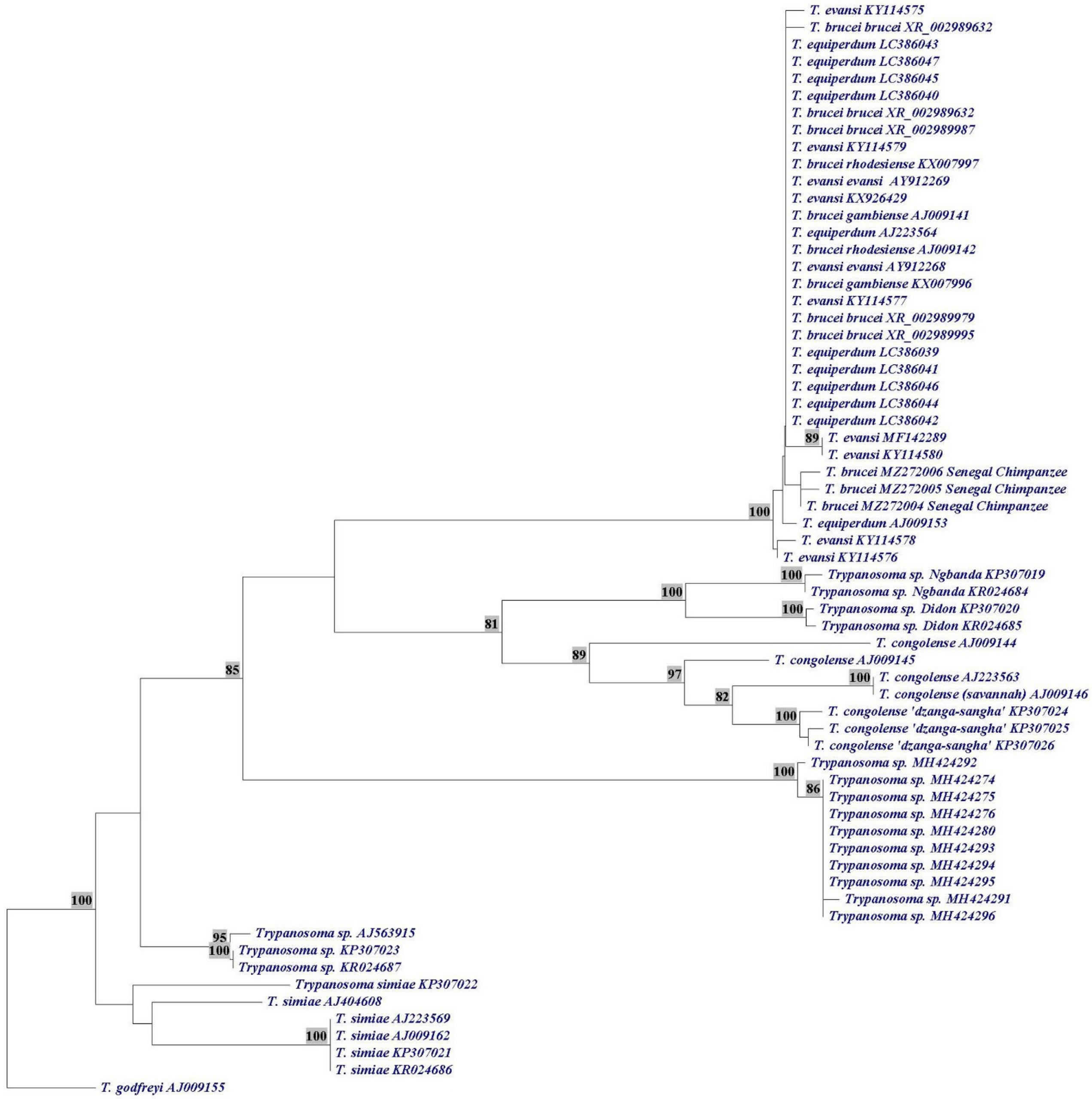

Figure S2. *Trypanosoma brucei ssu* rRNA phylogenetic tree. Phylogenetic relationships of the *ssu* rRNA gene (partial fragment) among *Trypanosoma brucei* sequences identified in free-living chimpanzees in the present study (labeled as Senegal Chimpanzee) and homologous sequences retrieved from GenBank (Sequence accession numbers are indicated). The analysis was conducted by the neighbor-joining method using Treecon software [96] after ClustalW alignment of the sequences [97]. Bootstrap values (1000 replicates) lower than 75% are not displayed. *Trypanosoma godfreyi* was used as outgroup to root the tree.
